# Supplementary material for: Targeted syndromic next-generation sequencing panel for simultaneous detection of pathogens associated with bovine reproductive failure
Source: J Clin Microbiol. 2024 Dec 10;63(1):e01433-24. doi: 10.1128/jcm.01433-24 (PMC11784112; doi:10.1128/jcm.01433-24)
Supplement: File S3 — BovReproSeq synthetic positive control sequences. [file jcm.01433-24-s0001.pdf]

Supplemental File S3: Bovreproseq synthetic positive control sequences

Synthetic controls were created and used to optimise and validate the targeted next-generation sequencing approach before testing it on clinical samples. Random sequences with equal nucleotide frequency were generated using the RSAT random sequence web tool (http://rsat.sb-roscoff.fr/random-seq_form.cgi) and then flanked with target-specific primer binding sites. The size of each synthetic control was designed to match the actual size of the target. Two to four of these synthetic controls were integrated into a pUCIDT plasmid containing an ampicillin resistance gene, creating a final total of 17 plasmids. *E. coli* DH5-alpha was transformed with these plasmids, and control stocks were stored at -80 °C. Subsequently, plasmids were extracted from overnight *E. coli* cultures in lysogeny broth (LB) containing 100 µg/ml ampicillin using Qiagen QIAprep Spin Miniprep Kit (Qiagen, Cat no. 27104) and plasmid DNA extractions were normalized to 5 ng/µl for further use. These plasmids were pooled and used as a positive control for our assay.
Each table below lists the primer binding sites for each target and each fasta sequence represent a plasmid with synthetic controls. The target-specific primer binding sites flanking the synthetic sequences for each target were highlighted in the same color.

Organism	Gene	Primer name	Primers (5'→3')	Size
Coxiella burnetti	transposon-like repetitive region	Trans-1-F	TATGTATCCACCGTAGCCAGTC	687bp
		Trans-2-R	CCCAACAACACCTCCTTATTC	
		RC	GAATAAGGAGGTGTTGTTGGG	
	com1	CoxF2	ACYGCAGGCGTGGCGATAG	689bp
		CoxR4	TGAAGGTTTTGTTGTGAGGTGGC	
		RC	GCCACCTCACAACAAAACCTTCA	

>Bovreproseq_synthpos_Coxiella_burnetii_1400
TGAGTCTTCGAAGTCACTATGTATCCACCGTAGCCAGTCCGTGATCGGGCCAAACGACGCTATTTCTTGGCCTTACTGCAGCACG
GGGTCCCTGACCATCTTGGAGTGAGTATGACACACAACTGGCGAATTCAAATGACCTTGCTAAAATTACCACTACCGTATCAGAT
TGTTTCGGAACCTTTCGAAGGTAATAAGAACCCTGACCACCGTTAATACTGAAGGTGCAACCATCGACAGATGACTCTGTCAAA
GTAAAAGTCCTTTAAAGGTGCCTCGTTAGTTCGAGCTTCGAAATGCCACAGCTATGGTAGGACAACGCTGTCGTCTCGAACGCG
GTCTAGCAGTCTCCTCACCAGCGCCTTATGCTTGATTCGTTCACCGACCTTTGTGTATCCAGCTTGTAGCCTAGGCTTTTACCCCG
TACGCCTGTACATGGTGTGTCCTAGGCTCCTCATACCTATAAACGACGTTCACAACAATGAAACCGCGACTTAGATCTCCTGTCA
CCGATTATTGCTAACGTGTGGCATGGGAGGTACAAGCCGAGAACGTGTAATTATCGTTCTGACCCGGATCACTTAATGAATAAG
GTCATTAATCATGGCTCACCCTTGATAGCCCGGCCATGACCCGTTGATAAGTCTGACCTCGAAACAGAGGGATTCCTTTAGCATA
AGCTCGAATAAGGAGGTGTTGTTGGGGGCCCACAATTGCACCCAAGTGAATCCACCGCAATCATTCTATCCTGCATCCCGATTT
GGCGGGACACTGCAGGCGTGGCGATAGAGCCGCAAATTTGTTGGCTCCATTTCCAGGCTGCGGGACCACTCTTGAAGTGCTAG
GAGGCTCAGATGCTCTTTATAGGCTGACAGACCGGCGGCTGGCATCCCGTTGACCCCCTCCGTTTCAGTGGTGGAATTAGTGGT
AGTGTGGAGGCTGAGTAAAAGGATTGTATGGAGGCGACCGGCGACTCGCTCAGTCACAGGCCATCACACTATTACCAGTCATG
CACTAAGAGCGTCTTCGGTGCTCGACAGTAACTGCCTGATCGCCCTTCCCCTCTCTTTTATAAGGGGGATGGAAGTCCGTAGTGT
TGATAGCCGCCCGTAACAGACAAGAGCATCGGCGGCCTGCCCGATTCCTTCTACAGTAGGTTTCTTCGTTCGTGATCACCTGCCC
CCGCATCTTGATTTACCTATTTAGAATCTCACCACCGGTCGTCTTAGCCTCGCCCTCATCTGGGGTCTGTAGTGACTATCCAGAGC
GTCCTTTGCCAACCTCTTGATCCCTAGGCTCAGATGGTACGCTTTACATGCCGACTCCCGGAGTTTTGCTCCACGGCTCCTCGCAT
GTGTCTGCGTGTGTCGCCTTAAGCCTTCGGACGCCCGCACGACTCTAGACCGTGCATACTGCATCTAAAAGCGACTGAAGAGAG
CCACCTCACAACAAAACCTTCAATAGCTACGGCATCTGTAGCGCAC

Organism	Gene	Primername	Primers (5'→3')	Size
Chlamydia abortus	Cabortus Helicasegene	Clone8-Heli-F	TGGTATTCTTGCCGATGAC	475bp
		Clone8-Heli-R	GATCGTAACTGCTTAATAAACCG	
		RC	CGGTTTATTAAGCAGTTACGATC	
Listeria monocytogenes	Transcriptional regulator	LM1-F	GCTTAATAACCCCTGACCG	260bp
		LM1-R	AATCCCAATCTTCCTAACCAC	
		RC	GTGGTTAGGAAGATTGGGATT	
	hlyA	hlyA-F	GCAGTTGCAAGCGCTTGGAGTGAA	456bp
		hlyA-R	GCAACGTATCCTCCAGAGTGATCG	
		RC	CGATCACTCTGGAGGATACGTTGC	

>Bovreproseq_synthpos_Chlamydia_abortus_Listeria_mono
TAATTATAACCGCTCCCGACTGGTATTCTTGCCGATGACTGAGTTGGGAAACGCATTAGCGTCGAATGCGTCTTGCGGGAGCTC
GCCCCTGTTCAATCTTGGTTGATGTCTTTCTCTAGGTCCTAGTGTCAGTTCGAGGATCCCCATGGGTAGATTTATATACAAGGCC
GTGTTAACGGAGCAAAAATAGACACGTGAACCCACCCGGTGACTATGAGGTGTCGGATACATGTTGACCTCCGTTGCTCGCATT
TGTGCTATCTTCGGGGCTGACCTAACTCGGGTGTACATCGGTGACAATCAGCCTTAGGTCAATCAGACTGTCTAGCTGGTGCGC
CGCAGAACTCCATAATAGCCAATAGTATTAATGTCTCTAGAGAATTTCAGCCACGCGCCATCACGTAACTATCCCCACTATGCGA
GGATACTCATAATGAGAGGTTAGCGCCCCGGGGTGAGTCCTCTTCTTACCTGGATCGGTTTATTAAGCAGTTACGATCCGGACG
TACGAGGGCGCGGCACAGGATGGCGCGCTTAATAACCCCTGACCGGTAGTGCCGCTACTGCATTCACAAGTGCTCAATTGCATC
CTCCCAGATACTTTTATTACCAGGTCTCCCGACAAGTTACTTCGAGTATTACTGTTCGCATATTCTAGTTGGTCTGGCGCGCGGA
GTGTGCGGCCAGGCGCCGTCGAGCGCTCGTTAAATGTGTCTAGCGTGGAAAAACAGCACTTGACGAAGTGCCATTATCTCTCG
GGCAGCTGAGAATGTGGTTAGGAAGATTGGGATTCCAGCAAAGGAGCCCCAATTCTGTGGATGTAGCCCTCCTGGTGAATTCC
GTGCAGTTGCAAGCGCTTGGAGTGAACCGAGGGTATGGAGTGGGTACGGGTAAACTCTTGCGACCGTGAGTCCGGGTGCTTA
AAGACGGGGAGATCGTGCAGACCAGTTTATTTAATATCTTACTTTACGCGAGGCTACGCGGCCCGGTCTGGCGGTTGCCATGCC
CATCACAGTTTGTCCAAGCTTGGATGGCCTTGACAAGTGGTATTCGCTATTAGCATGGGAAGCCCCATAGCTTCCTGCAGTGAT
GACAAATATAGTCCATACCTAGCTGAGCATGATGCGACCTCCTCAACGTTCTCCCGTATCTATGAGGATCACGGTGATGTTGTTA
CCACAGGCCTTCAAGATTTATGCTGGCTCAAGGCTCCCTGCGACCAGCAAGTCGCGTCCGTGAGAGCGATACGACCCCGATCCC
GTGAACTGGACCTCCGATCACTCTGGAGGATACGTTGCTCCCTCGCATAGGAATGACGTTAGT

Organism	Gene	Primer name	Primers (5'→3')	Size
Ureaplasma diversum	16srRNA	UDF1-	AAATGTCGGCTCG**C**TTATGAG	311bp
		UDF2-	AAATGTCGGCTCG**A**TTATGAG	
		UDF	AAATGTCGGCTCGMTTATGAG	
		UDR	TATCGATAGATAAATTAACTAGCG	
		RC	CGCTAGTTAATTTATCTATCGATA	
***Ureaplasma* spp.**	16srRNA	UGPF-	GGATGAGGGTGCGACGTATC	642bp
		UGPR-	GCGTTAGCTACAACACCGAC	
		RC	GTCGGTGTTGTAGCTAACGC	

>Bovreproseq_synthpos_Ureaplasma_diversum_1400
ATTTGGTTTACGCTTGTGAAATCGGCGGAAATGTCGGCTCGCTTATGAGTCTGCCCAACGCGATCCGTACGAGGTCAGATGACA
GGCCCCGTCTTCGTTACCCGCCTATCCTGTCCACCATATGATGCTTCCGGTAGAGCGGGTGGCGTTAACCACAATTAAACGATTA
GGTCGCTTGAGTACGTACCCCTACCCGCGGCTTGAATACCTAAAAAATGCCCCTACAGACCTGTAACGCGATGTAATTTCGCAAC
TTATACACGCCCATCAAAAACAATAACCAAGAGTCGGGCTGGTCGAGTGACCAAGTGCCGACGCTAGTTAATTTATCTATCGAT
AGTGGGCGGGCCGCATGGTGTGCACACATCCAAGTCTTTTCCGCAGTCCCTAGCGCCGTCGAGTATCGCGGTATGACCTGGAT
GAGGGTGCGACGTATCGTGGGAAGGGTGCGTGAACAGAATTTAAAAGGGACCCGACTTATATGTCCCAAAGTGTCGCGCGGA
TGGATCTTAGTCCAGTCGTTCGGACCGCGAGGATGGGATAATCATTCACGCCCTGCCCACTTGTTCGCTCCGTCCTGCTTATTTA
CCCTTACATCTACCCAAAATACAGTGCGGGGAGTAACACTATCTGAAGAATTTGGAATTTTGGGAGTAGGCAACCGGCACACGC
CCTAACCGTAGTGTATATTTACGGATTCGGGTCGATCGGCTACGATATTTTAGGACGCTTACCCGTGTCCCACTGTAGCAACTGC
AGTGAGTTGGACTTGCTTAGTAGGAGGTCTTAGCTCAATCACACCTGTTTGACCAGCAATAGTTAACATCTCGACACATGGCTCA
ACATTATAATTTGATTGCTTTTAAAACATACGACAGAAAGTAGTTCCGACTTGATGATGCATGACCTTCTGTGAGTACTCCAGCTA
CGAGATGCCTTTCGCCCAGAGTGCGAGAGTTTTTCGTCGTACAAGGACTTACTACGCCACGTCCAATCGAGCAACATATGCGTC
CAGTGTCCACCCTATGAACCCGAGTCAGTCGGTGTTGTAGCTAACGCAGTGCACCGACCTTATGTAAGTAAATAC

Organism	Gene	Primer name	Primers (5’→3’)	Size
***Leptospira* spp.**	16SribosomalRNA (rrs2)	rrs2-F	CATGCAAGTCAAGCGGAGTA	541bp
		rrs2-R	AGTTGAGCCCGCAGTTTTC	
		RC	GAAAACTGCGGGCTCAACT	
	LipL32	Lau01	ACTCTTTGCAAGCATTACCGC	660bp
		Lau02	AGCAGACCAACAGATGCAACG	
		RC	CGTTGCATCTGTTGGTCTGCT	

>Bovreproseq_synthpos_Leptospiraspp. _1400
CCCCAATCAGAGTTGATATGAGGCATGCAAGTCAAGCGGAGTAGGTTGACCACTAGTAGTTTCTAAGTGGATACCCACCGGCA
GCATTGTAGGGTACACGGGGTACGGTAGGTCTACGTACGTAACCCCGGTGTTATTAACTGGACTGTTCAATGGGGACGATCTAT
TTTCCAGCGTCTCATAACCGGACTAACTCAGCGGGGGCACAAGGCACAGAGTTATACGCGTATCTAAGCTGTCGCGTCGCAAGG
CAACTTAGCAGATTACCGGCGTAGTGGATACGTTAGAGGTAACGGGCCTTAAGACGGCGCGTATGGTGATACACGGCGACAGA
AGTCTTTGGCCAGGCGGCCATCCTTCTAACTAGGTAAATTCGACTGAGTCTGCCAAAAGGAGTTGCGTGTCCTTCCTGACCGAG
GGGTTGTTCAACCATGCCCGTACGCGGTCTACCAGCGTGTCCTTCAGCGACTGCAAGCAGTCAAAGCTTCTCTGCGCGCCATTA
ATTGATTAAAGAAACGCCAAGTACATGAACACGGTTCCAACAATAGAAAACTGCGGGCTCAACTTATGATGAAACGGAGGGAA
ACTGAACCTAGCAGCGCCCAAGACTCGTGACTCTTTGCAAGCATTACCGCTTACTCGCTTGTGGTTGTGTTGCTAGTAGGGTAG
GTCAATGCGTATGCGGAAGTCGCTGGGTATGCTCAATCAGTTCAAGGCCTGTGTCTTGACGTTTTCACGTCACACTGAGGTGAG
CGGCTGATAGAGCGAATGCTCAAATGTCCAAGAACGACCCTGCGCGGTCAAAATAATCCTATAGAGCTTAGGATGATACGGCA
CATTACCGGGGGGAAGAGCGTAGGACGGCGACCTTGCTTTTATCGCGTACCAGATATCTTAGGGTCTCGACAACTAACTATCTG
CGCTGTATTCGTAGGGCATGTCTGCAAGTATACACAATGTGAGCCGCAGAAAGCCAGCACGAGGCCCGCTTAGTTAGCGGCTG
TGGGTACTTGGCTACATAGAGGTATAGGCTGTACAATCTGGTATCGATGGTACGATGTAAACCAAATTTCGGCGGTGTCAGCGC
TCCGGCCGCGGGACCTCTGCAACTCAGAAAGCATCAGATGTATTACTCTTCAATGAATCGCTATGGGGCATCTAATGACAAGAG
GTATAGAAAACATAAGCACGGGCTCACCGTGCGCAATGGCGATGGTATATCCCCTACTTCGGAAGCGACGCCAAGGCAAGCGT
CGTTGCATCTGTTGGTCTGCTTACTCCATCCCGGGGGTATTACTGGTGGCCTAATTT

Organism	Gene	Primer name	Primers (5'→3')	Size
Neospora caninum	pNC-5gene	Np21	GTGCGTCCAATCCTGTAAC	328bp
		Np6	CAGTCAACCTACGTCTTCT	
		RC	AGAAGACGTAGGTTGACTG	
Tritrichomonas fetus	ssrRNA	TFR3	CGGGTCTTCCTATATGAGACAGAACC	347bp
		TFR4	CCTGCCGTTGGATCAGTTTCGTTAA	
		RC	TTAACGAAACTGATCCAACGGCAGG	

>Bovreproseq_synthpos_Neosporacaninum_Trich_fetus_1400
AGCTCAGAAGCCTTAAGTCTATGTGCGTCCAATCCTGTAACGGAGCCCGTATAGATATATCCACGCTACGTTGGTCCTAGCAAG
GGGTCCCTAGAAGTCGTGGTTTGAAGCTTAGGCTAGTCCCTCGCGTAAAAAGAAACCTACTCGTGCCGGCTGAGTACACGAGC
CATTTCTGCCCAAATGCTCATGTCGGCAAATATACCTCGCCATGCTCCTCTCGTAGTGCATGTCACCTAATATGAATATTCCGTTA
CCGTCCTGCAGTGTTTTTCTCCGGTTAGGAGACCAAGTGAGCCAGCGTAGCCTACATTGCCTAGCACTCCAACGCTCGAGAAGA
CGTAGGTTGACTGTGTCCAATGGGTACTCGCAACTGGATAAGTGGAATGGAGCCTGATAATCGGTTCTGAGGTCGCGAATTTTC
ATCCGGGTCTTCCTATATGAGACAGAACCATATGACTCCCCGCACCCACATGACCCTGTATACTGCCTCGGCTGAGGTGGGTTTC
CAGCTCTCTTGGCAATGGACGCTAACGAGTACCGAGATTGCAGTCGTTCAACTCAGGCGCTACGGGCCCGCTTACCGTAATCGG
CGGGTCGACTGATGTCCCTGGCGGCCGAATAATCCCGTTAGCACAGTTTCGGCTCTAGGCTTGGCGCAACCCTTGCGGTCTCGG
TGAATTCCCGTGTATTGTGCTCGATCTCCCCTCCAATCCACAAGATACCACATGATCTATGATTAGGTCACTTTAACGAAACTGAT
CCAACGGCAGGGTCAAGAATGCTTCAGTGT

Organism	Gene	Primer name	Primers (5'→3')	Size
**Bovine Herpes Virus Type1 (BoHV-1)-IBR virus**	gBN-1	gBN-1-	TCTCGACCGGGGACATTATC	385bp
		gBN-2	GCCTCTTCGATCACGCAGTC	
		RC	GACTGCGTGATCGAAGAGGC	
	gE-1c	gE-1	GCTTCGGTCGACACGGTCTT	268bp
		gE-2	CTTTGTCGCCCGTTGAGTCG	
		RC	CGACTCAACGGGCGACAAAG	

>Bovreproseq_synthpos_IBR-BoHV-1_1400
AAGCTATAGATCCCGGGTGTCTCGGGCCGATATCTCGACCGGGGACATTATCTGATCTAAGCTACCCACTCACCTGCAGGCAAT
ACATCCCGGGTGATACGAAAACTTCACCCGCGGCAAACAGGACTGCCGCTAGTGTTTAGTGGGGAGAGAGCAACGAAACACAC
GGTTTTACGTTCATTGTTCCGGCAGTACGCATCATTGTAACGTGCGGCTCTCATCAGAGTTGCAGTGCGCAAACCCGCGCTCGAC
ACCCTACCGCTACAACCGCACTTCACGAAATCCAAAAACCTACTTGGCCGCAAAATTATGTATCGTAGATTGCGGCGGTTACGGC
TAGTAACGTAACCAATATCAGTGCCTCGGTCTTGTCCTGACAAGAGCCAAATTAGCTAGTGACTGCGTGATCGAAGAGGCTTAA
TGGGGTAACAATGGAGCACGCGCTGTAACTGAGGCTTCGGTCGACACGGTCTTTTTATTGTAGCGCACGATACTGTTACCCTAT
TACGTAGAATGGTACGCAAACTGTGGAACGTACAGATGTCGAGGCTCTGCTCATCGTAGTAAGGTGGTGCATTGTTGTCACCGA
GAATGGTACAGCACGATACGTTGATCGTTGTCAGGGCGGTGGAGCGCTTGAAGGAGTGCGTAAAAATTCAACAAAGGCCTACT
CACGCTAGTGAAACAGAGGCTGCCACCATTCGACTCAACGGGCGACAAAGCTCGGGACCATCCAACCTCGTCTATCTGC

Organism	Gene	Primer name	Primers (5'→3')	Size
BVDv	5'UTR	PF1	ATGCCCTTAGTAGGACTAGC	285bp
		PR1	ACTCCATGTGCCATGTACAG	
		RC	CTGTACATGGCACATGGAGT	
	Npro	B32(Forward)	CCATCTATRCAYACATARATGTGGT	441bp
		B31(Reverse)	TGCTACTAAAAATCTCTGCTGT	
		RC	ACAGCAGAGATTTTTAGTAGCA	

>Bovreproseq_synthpos_BVDv_1400
CTATTAGTACCAGCTTCACACGATTCAGTTAGCCGTCGATCAGATGCCCTTAGTAGGACTAGCCTACCTTTACAATCAATACTATC
GTCCTATACTCTTCACAAATATGCGTTGTACCAGGGTGGACGTTTCTTCAGTGACGCGTATTCCTACTAGGTCATATGGTCTCCAT
TCCGTGCGCTAGATACGGTCGAGATGGCGTGATCTAAAGTTGTGCTAAGGACAGTATGGCTTGGCGCGTCGGAACAATGTAGT
TTTAAGAGTACTCATGCGGAAGTAGCTTAAGCTTCAGTGCAAGACTTTACTTCCTGTACATGGCACATGGAGTGACCTAAGGATT
ACTGTTGTGGATGACCCTGCGTGAATAAACG**CCATCTATACATACATAGATGTGGT**GCGCAGAATCACCGCATCATAACCGAAG
ATGAAACAGCACTTGGGGGCTCCAGAAACGTAGGCCTCCGCCGGACTGCCGCGATTTCACAGTGCCGATTACAACATATCTCTG
ATCCTTAGGATGTGTGCCTAGGACCCTTACTCACTTGAGCACAAACTGAACATCTGCGTTGCTAAACGAACGGACCACGTTGGC
GTTACCTTTCCGCGACGACGCAGCCGCGGTGACTGTTTTAGCCATCTCGAGGTCGCGATAACTTGGTTGTGGGGAGCTACTTTG
GTTCATACCACGCAACACCGTACAGCGCGACCAGATTCCACAGCGCGGGGATTAGGACGGTAAAAAGGTAATCTGGAAGTTTT
GTAGGCAGGTTGATCCTTGTAGAGGGGATAG**ACAGCAGAGATTTTTAGTAGCA**GGCGAATTATGATACCTTCACCTAGATCAGC
ACAGGCACGC

Organism	Gene	Primer name	Primers (5'→3')	Size
Campylobacter fetus	nahE	nahE-CFETSpp-L	GGTTATTTTTTATAACTGTAGGAATGCAGAT	390bp
		nahE-CFETSpp-R	GATCGCTTAAATCTTGTACTTTTAGCTTTT	
		RC	AAAAGCTAAAAGTACAAGATTTAAGCGATC	
Campylobacter fetus. Subsp. *fetus*	sapB2	CF-F	GCAAATATAAATGTAAGCGGAGAG	433bp
		CF-R	TGCAGCGGCCCCACCTAT	
		RC	ATAGGTGGGGCCGCTGCA	
Campylobacter fetus. Subsp. *venerealis*	*virB11*	*nC1165g4F*	AGGACACAAATGGTAACTGG	233bp
		nC1165g4R	GATTGTATAGCGGACTTTGC	
		RC	GCAAAGTCCGCTATACAATC	
	parA	*VenSF*	CTTAGCAGTTTGCGATATTGCCATT	142bp
		VenSR	GCTTTTGAGATAACAATAAGAGCTT	
		RC	AAGCTCTTATTGTTATCTCAAAAGC	

>Bovreproseq_synthpos_Campfetus_CFF_CFV_1400
AAGACAAAAGGGATTAGTGGGGTTATTTTTTATAACTGTAGGAATGCAGATTCAGACACAGGGTTGGCTTATTTGCGTCTGGTA
TGTCTTTAACAGCGATGCTATTTAAGGCACGAAGGGAAAGCAGCAAACCCTTTCTAACGCTAAATACCTGTGATATAGCCAGCTT
ATACTAGTGAATGTCCCCCCTTGCTAACGTCGTATGCGTTTTGCGTCCATTGCGATACCTACAAGGTAGCGCTTTATGCTTCAAAT
TCTGGGGAGCGTGGCATACGTACGATACTGAGACTTTATCCAACAAGATGGGGGCTCCACGTGTTTGGTGATTAGAAAGGAGC
GACTGGATGGCAAGAGTTACACGATATAAGGCCGGTACCATCTAAAAGCTAAAAGTACAAGATTTAAGCGATCAGATGCCACAC
CATGAAAAGATGCGTACGTGGAGGACATGTGGCAATTGTTCGCACGGTTGTTATGTTCAGCAGGCTGAACAAGACATGGAGGG
GTATTTTCAAGGCGCATTAAGTTAGGTTCCGTCGTTACCTGCAAATATAAATGTAAGCGGAGAGGCGGCTCGTTCGGTCTACAG
ACTATGAACCGGCAGGATCTCCTAATCTCCCTCAGCTCCATCGACTCCGAATTTCGTAAAGTTCAGACATCAAGCGATTGCAGGG
ACCAAGATCATCATAAAGCAATTTTCAGTAGACCGTCGTATTCTAAACTAGAAAATATCCTCCCAATGGATGGATCCCGGAGAAG
ACAGGCGTAACCGCATGGAGGAAGAGTCCTACATCCTCACTGTTTCACCTCATTTACGACCCAACGTAAGGCAGTAGGCAGAAG
TTACCCTCCCCTGTGCAATCAAAGCAACGCTTATGGCATAAAGAAGCCGTTACCATAACGATACTCGTCTTAACCTTTGGCCGTTT
CCGGCCCTGCGGCTTGAGACCACTTACCGGATAGGTGGGGCCGCTGCATATCATAAATGTATTGACAGGATCGGTGCGATTAG
AACCTGGGTTCCCTTTTAGCCGCACTACACGGACCAACTCAGTGAAAAGAGAAAGGACACAAATGGTAACTGGTGACGTGGGC
ATTCCGTCACCTCAAAATGGTATGCAGATGCGGTGGGACTAAGAGCCATTATCTATGATGTGTGCTGCGAAAGCAGTCTACGCA
ACCGAGGCCGAAGGGACTGGCAATGCAGGTCTGCCAGGTGCGCGCGTGTGGTACTAAGGTGTCTTACTTTATTAATAGTCGCG
TACGCAACAGCGCGCTCTTGGAGCAAAGTCCGCTATACAATCTATCACCGTGTGATCTCGGGAGTGACGAGGGGGCATTTTTTA
TTATGAAAGAGGCAGAATGCTATCGCCGGTTCAGGGGCATGATGAACGATGCCACAACATGCGCCTTAGCAGTTTGCGATATTG
CCATTCCAACCTGTACGTGGTTGAGGATAGAAAGATAACTCAATGCCCGGCAATCATTCGGGAAATGACCGCAGCCTCGTATCG
CTGGATGGAACAAAAGCTCTTATTGTTATCTCAAAAGCCTCGCGCACCGTCAACTACAACCCACCACT

Organism	Gene	Primer name	Primers (5'→3')	Size
Campylobacter fetus **MLST**	*aspA*	*aspA-F*	CCTATGACTTTAGGTCAAGAG	477bp
		aspA-R	TGTAGCTAGAGTACGGCAAG	
		RC	CTTGCCGTACTCTAGCTACA	
	glnA	*glnA-F*	GATGGTAGTTCTATAGACGC	477bp
		glnA-R	CTTCCGTTATCTCCATAAAGC	
		RC	GCTTTATGGAGATAACGGAAG	
	gltA	*gltA-F*	CGATATAGCGTGGCTAGCTG	402bp
		gltA-R	AGCGTGAGTAGATCCTACG	
		RC	CGTAGGATCTACTCACGCT	

>Bovreproseq_synthpos_CampfetusMLST_aspA_glnA_gltA_1400
CCACCCAGGCGCCTATGACTTTAGGTCAAGAGGAATGCATGCCCATTGCACACGGGCCCAGATAGTACGGAAGGATTTGAGGA
GCTCAATTCTGATTGAACTGCGCCGTTAGCGCAGATCGTACTCCGTGTCTATCTCCACTACCACATGTTCCGAACTAGACTGACT
GATTTACCAAGAGGAAGCAATATGTTATAAACTCATTGTGTTGAATAGCGGTAGGCGTAGAACACAGTCAATTGATTTATTTTTT
ATTGTGCCTGTAAGGCTTGCGTAGACTACAAGTCGGGTCTTTCCAGAACGGTGCGTATCAAACTTCAGCGCAGACTAGGCTACA
GGAAGAAGCGAAAGACCCCCCTAACGCAAACTGTATGCAGTCTTTGTATGCTCACACTCCCTCGAAGTCTCACAATGTCCGGGC
GGTTCCCGAGTTAGAATCAAATCAGCGGTCATGTACCGTGGGCTTCCCTTGCCGTACTCTAGCTACACTCGTCTGAGCTAATTCT
GAGTTAATCGATTTGCACGTTACATCAATAATGTGATGGTAGTTCTATAGACGCGATTCAGGTCACCACTCCATATGTTACCCACT
CTCGTGAAGCGTCAAGCAAGAGTTGTCAATTCGGTATAGGCTCGTGTGTCCGTGTTATTGCCGCATGTAAAGCGGCAGCGCCGC
GGTTGATTGAATAGTGTGCCGGGGCTGGCAGAGTGGTTGGAGCGCTCTAGTCATAAGGATTGACATGCGATAATAGGTCCAAT
GCAAGGTGTTCGGACGTTATCTCATTCCAATGCAATGCGTATCACATGCTCGTGGTGAGTGGGGCGATGTACGACCTGTTGGTG
GCATTCATCCCGGGGTTAAGACGCTCCCGTGTCCAACCCCATCCCTTAATTGAGTTGCGGTTGCTTTCGCGCCCAAAAAAGCCGT
CCTCCAACTCCGGGCAGATGGAGTAGGTTTAACCAGATTCTCCTCAGAAGTACCGTTACGAGCTCGCTGCTTTATGGAGATAAC
GGAAGCAAGCGTACCCGCCCGTCGGCGAACCGTGAAGCGATATAGCGTGGCTAGCTGGCTTACAATACTACGGCAAACTGGCA
AGGTCCCAACAACTCTTAAGCCCAGCCGTGCCGGTAACGAGACTCGACGGCGTTCAATCATCGAGACTGGCTCTCTCTTGTTGG
TGAGTCGACACACATGCGGGATCACTCGTCAACTTTCACGCTACTTAGCCTCTGGGACCTTCCCAGGTGTCCAATCCACCTTGGA
TGAGGGATATGCTCGACCTCGGGCCTCGGAGATTCTTGGTGAAGTGACGGTTCTGACAGCCGATGTTATGTGAGTAGGATTTTG
GTCTTTGCCGGTGTTGCATATCCTGGACGCGCTGAACTTAGAGGCCCCCGCGCACACGTCAATAGAACCCGGGCCCGGCCGTCC
CGTAGGATCTACTCACGCTCAAATTATGCACTATCTAGATCTACGCGGCTGAAGAAGTAATTATTGACTAAGGTGATGAATTACT

Organism	Gene	Primer name	Primers (5'→3')	Size
Campylobacter fetus MLST	*glyA*	*glyA-F*	GATAAAATACTTGGTATGGATC	507bp
		glyA-R	CCCTCTGTTTATTAAGACTTC	
		RC	GAAGTCTTAATAAACAGAGGG	
	pgm	*pgm-F*	AGAGTTGTTTTGGACGTTGC	501bp
		pgm-R	GTAGCTCATCAAGAGGTCTC	
		RC	GAGACCTCTTGATGAGCTAC	

>Bovreproseq_synthpos_CampfetusMLST_glyA_pgmA_1400
GAAGCTTATGTTCATGTGATAAAATACTTGGTATGGATCAACACATCCCCGTTCGAGGTCAAAGGGCGTGGTTTTCGGAGTCTG
TGACATCTTTCGCCGGTAGTCATTCGTCAGCCATATGGCTACTCTGTCCTCGCCGAACTGTGTATCCCACCCCACGAGACGCAGA
TCATGATCCCTCTTGGGAGGGAGGTCACATGTCTGAACCCTGTACCGGATCGAAAGACTAAAACATAGAGAAACTTTCGCTGGT
TCCTGCGCACCATCGCGACTTCAAACGTAAGCTGTTAAATCCGTCGAAAAAATAGCTCTTGCGAGGCCAGGCTGCGCGGCCTGC
ATTGGGCATATTGTGAATCCGATAGCTGTGAGCTTGACATTTTTCCGAAACGTAAGCACTCGCTCGGCGTTCGCCGGGACGGGT
AAAGAAGTGGGTCGATGACCAAGACTGCAAGCAGGAGGACAGGCGCACATCTTTCATTGGGGTTTATTGGCGGTAGTGCTTCG
AGATCCACTCTTCTCCGGAAGTCTTAATAAACAGAGGGTTGGGTGTTTTAACTCAGCCATTGCTTCTACGGCTACTTCGGGCGTG
CGCGAGGCTACTTCGACTGGAATGTAGGAGAGTTGTTTTGGACGTTGCTGTCCAGTTTTGGAGATTCTCCGCTGGCCGGGGTG
GCACTACACTGTTCAGTCCTGTGCTCACCAGATGTGCATCATCGGACTAGGTAGATCTTGGTCCCGGGGAACCAGCGTAGGATT
CTGGTTGGTGCTGCTGACCGAGGCGGCCATACCATTGGGTCTAAAAAGACACAAGGTGCTCGTATTTTGCGTCAGGTGATACTC
GTCGCTCCCACTTGGCGTTGTACACACGCAGCATATCGATTTGTCAGAGGTGTCTCGAGGTCATATACCTATATATTTAGGGGTT
GTACTGCCACTGCTAGATTACCGGGCCTTCTCAGAGCCCGAGTGAGTCATGTGCCAAAGTTCTGTGTTTTTCGCGGGAGATATTC
GTGGTCCCACGGTTCTCGCTAACGGAGGACTGACCGCCGCTTAGCGTCCACCGTAAAAAGATGCATATGAGCGGGTAATGGGT
GGGCAGTTTCTGAGACCTCTTGATGAGCTACTTTGGATGGGGATTATAGGTATCCAA

Organism	Gene	Primer name	Primers (5'→3')	Size
Campylobacter fetus MLST	*tkt*	*tkt-F*	GAGATAGATTGGTATTTAGCGG	459bp
		tkt-R	GTGACTACCTTCTAAATCTCC	
		RC	GGAGATTTGGAAGGTAGTCAC	
	uncA	*uncA-F*	AAGAGTACGGTGCTATGGAC	489bp
		uncA-R	CTCTCATCAAGATCGCTTGC	
		RC	GCAAGCGATCTTGATGAGAG	

>Bovreproseq_synthpos_CampfetusMLST_uncA_tktA_1400
CGATACACAATGGATCTCTGACTTCCGGAGGCAGAGATAGATTGGTATTTAGCGGTCTAATGTCCAAAATAACGCGGATCGGCA
CATTAGTAGTATTTCTCAAGTTGGCTCAACGACAGAAATTCGCCGGCGAGGATGTGGCCTGTATCCAGCAATCGGCTCGAGAAT
TTTGACCGCACGTCCTAACCCCGTCTCTCAGTATCGACACCTCGCTTACCAAACCATCAGGGAAACCCTTAGACAAAGGTAAAGG
ATCTCCCCGTTGCGTCATCAGTCCCAACAGATATGATGTGCGGTTGTCCGGACTCTATGCGGGAGAAACTGCGGTGTGCTTCCG
ACCCTTTGCTGCTGCGTGCTTAGGGGGACTATTAGGTAAAAGTAGTCCAGATTTAGGTACATATACATGATCAAGTTCAACCGAT
GCCCTACAGCCATAGCAGTGCCTAGTGCTCAGGCTCCATGTTACTAAGCCGTGGAGATTTGGAAGGTAGTCACTGAACAAATGG
CGTGCTGCCCTGAGTACTGCGACGGTCTCGCGGAGATAAGAGTACGGTGCTATGGACGTGCTATTCGGTGATTAGAAGGGTCG
GGCCTATAACGAGTGTACCAATGTATACAGCCGACATAGTTGTCAATGAGCCGAGCGTCATCTGTAATATGCGTTACGGCCCTG
CGGGCCACGTCCTAGAAGTATGTGGTTCGCTAGTGCAAGGTCGTGATTTAAACGATCGCCCACTTCCCGCAGCACGCCTAGTAG
CTTTTATTCAATGCTAGGGGAGGCACGTCATGCGGAAAGCCGTATGTTTCGTCGTGTTACTTCTACAGTTTACTTATAAGTGCGG
AGCCCTGTTCGTAGTGCGGACAGCTTACAATGCGGTTGGAAATGCGCTGGGACAAGAAGTACCTACATCGAGGATGGCTACCG
ACTATGAGATAGTCCAATGTTGCGAACTTTGGGCCATGTCAAGGCGCAGGCGTTACCTAGGAGATCCGCGCGCGGCAGTATCC
CAGCGCAAGCGATCTTGATGAGAGACTCTCAGTGATCGAGGATGCCTTAC

Organism	Gene	Primer name	Primers (5'→3')	Size
**Internal Control Enhanced green fluorescent Protein**	IC-EGFP	>Egfp-F	GACGTAAACGGCCACAAGTT	550bp
		>Egfp-R	GGGTGCTCAGGTAGTGGTTG	
		RC	CAACCACTACCTGAGCACCC	

>Internal control enhanced green fluorescent protein gene
GACGTAAACGGCCACAAGTTCAGCGTGTCCGGCGAGGGCGAGGGCGATGCCACCTACGGCAAGCTGACCCTGAAGTTCAT
CTGCACCACCGGCAAGCTGCCCGTGCCCTGGCCCACCCTCGTGACCACCCTGACCTACGGCGTGCAGTGCTTCAGCCGCT
ACCCCGACCACATGAAGCAGCACGACTTCTTCAAGTCCGCCATGCCCGAAGGCTACGTCCAGGAGCGCACCATCTTCTTC
AAGGACGACGGCAACTACAAGACCCGCGCCGAGGTGAAGTTCGAGGGCGACACCCTGGTGAACCGCATCGAGCTGAAGGG
CATCGACTTCAAGGAGGACGGCAACATCCTGGGGCACAAGCTGGAGTACAACTACAACAGCCACAACGTCTATATCATGG
CCGACAAGCAGAAGAACGGCATCAAGGTGAACTTCAAGATCCGCCACAACATCGAGGACGGCAGCGTGCAGCTCGCCGAC
CACTACCAGCAGAACACCCCCATCGGCGACGGCCCCGTGCTGCTGCCCGACAACCACTACCTGAGCACCC

Organism	Gene	Primer name	Primers (5'→3')	Size
Toxoplasma gondii	Major Surface antigen	Toxoplasma_gondii_DS38	CGACAGCCGCGGTCATTCTC	550bp
		Toxoplasma_gondii_DS39	GCAACCAGTCAGCGTCGTCC	
		RC	GGACGACGCTGACTGGTTGC	
Sarcocystis **spp.**	18SrRNA	Sar-F1	GCACTTGATGAATTCTGGCA	609bp
		Sar-F2	CACCACCCATAGAATCAAG	
		RC	CTTGATTCTATGGGTGGTG	
Pan coccidia **spp.**	18SrRNA	Coccidia_18s-F	GTTGTTGCAGTTAAAAAGCTCGT	
		Coccidia_18s-R	ATCTAAGAATTTCACCTCTGACAGT	
		RC	ACTGTCAGAGGTGAAATTCTTAGAT	

>Bovreproseq_synthpos_Toxo_gondi_Sarcocystis_sp_Coccidia
TTGGTTATGGGTTGTTGCAGTTAAAAAGCTGGTGGCCAAATTACTTCTCGTCTTTCCACCCACGTGGATCTAATCGGATGTGGTC
GCTTCTACCCCGTTGCACCCAAATTCGCAATATTACTATATTGACAGGAGTATCGACATAAGGTGTCGGTGTCCGGGAAAAGGG
CGATAGATTGGCAATCTTCGGGCTCGGATACGTTAGCCCTTTAATTCAGACCTATTGAGTCCATGGGTCATCTCCCGCTTGCAGC
CAATAAAGCGGGAGGAAAGTTTTAGCTACCCCAAGTCGTATCATAGCCTGCACTGTCAGAGGTGAAATTCTTAGATCTGAGAAA
GACACTAGTACGATGTGCACTTGATGAATTCTGGCAGCCCTCTCGGCCGGGGACGTGTGCACCGAGATCTTATCTTATTTTAGCT
TACAGACTATCGCACCACTCTCTTGGCTGTAGTAGGTTACACGTCGCACTCGAGGTCGCTCAGGTTTCTTCGGTCGCTGACGTGT
TTATCAAGACATATACCTGATTGGATCGGACGAGACGTTCGGTAGCTAAACGGTTCTGCGAGTAGACAGCGAGAGGCCCCACCT
GATGCAAACCTGATGGATGGGACGTTGGACTCAGTGCCGATCGATTGGCTTCTGTTGGTCTGTACCACGACATCGTTGGGACAT
AATTGCAATAGCGAACTGAAGATGCCGCTCCCCTTTCCCCGCGGCTAGGGGCCAAAACATCTGATATCCTAAACACAGTATAGTT
TCTGCAGTTGAAAGTCTGCGACCGACTTCGTTAGTGATGGCCGCCTAGCGCGGGGGCACGCGGGCTAGAGCGTACCCCCCGGC
TATGCCCGTTCCCACAGTCCTTCTCGGCATTATGTCGGTGCTTAGAGTGGGTACCGATATCACCGGTTGCTATCTGCCTTGGCTA
CGCGCATATTCTAGGCTTGATTCTATGGGTGGTGATATTCTAGGAGGTGGGAAGGTCGACAGCCGCGGTCATTCTCAACTGTTG
TTAAGCAAGCCATGTCCAAGAGCGTACCCTCGTGGAATAGGTGGTAACAATTGTTGCAGACCAGTCTTCTGCTCCGGCCTGCCC
TATCCGTGTATAGCATGGCTCATCAAGGTCAGTCCCATTACATAAAAGTGATACTTTTGTGGCCTGTTTCTGGTAGCCTCTGCGG
CCAGTCCTTTACAAAGATTATAGGTCGCAGTTTATGCAGCTTAGGACATGAGCGGTGTGTAGATCAACAAGCTCCTCCCTCGCCT
GGAGAATTGAGACATGGATTCCTTAGACGATTAAGGTCCATGCTTTATCAACGCTCGGCTGATTCAAGGGTCGTCGCGAATAGT
CAGAACCGATCGCGATTCACAATATAATTAGTGGATATATTGAGGATGGCTCCGATCATCTCGACGAATATTAAGACGAGGCCG
TCCTCGAAGGTAACGGAAAATTGCCTACCCAAGGACGTGGCAGTCACTATCCAAAGCCTGTTTGCTTGCGATGGTCGCAAGGAC
GACGCTGACTGGTTGCCTTCCGTATATGTGAA

Organism	Gene	Primer name	Primers (5’→3’)	Size
Bacillus licheniformis	adk-adenylatekinase	Blich-adk-F	GGTAAAGGGACACAGGCTGA	613bp
		Blich-adk-R	TCGAGTAAAGGCTGGGTTTG	
		RC	CAAACCCAGCCTTTACTCGA	
	gyrB-gyraseB	Blich-gyrB-F	AKACGGAAGTGACGGGAAC	518bp
		Blich-gyrB-R	AGAAACTTTTCNAGCGCTT	
		RC	AAGCGCTTGAAAAGTTTCT	
Tritrichomonas fetus	18SrRNA	TF-forward_18S	GTAGGTGAACCTGCCGTTG	330-360bp
		TF-reverse_5.8S	TTCAGTTCAGCGGGTCTTC	
		RC	GAAGACCCGCTGAACTGAA	

>Bovreproseq_synthpos_B. lich_gyrB_Adk_Trich_18s
AACCCTCACTATACGGAAGTGACGGGAACGTCTTCTAGATTTTGAGCAACGGAGAAAGCCTGTGAATGATGTCAGGAATGGCTT
ATCAGGGTAGGTCCTTCGCGTATATGTTGGACTTGTCTCTTATGCTTAGAACACGCTAATGCATACCGACCAAATAACGGAAATT
TAGTCGGGGGCGTAGCCCGCGTTGATGCATCCGGGCGGATTTAGCCGAACTCAGATCAGGTTGTTGCGCACGACATTCTTTATC
CCGAGCTCCTTTGTAGGTCGCCGGCTACCAATGGTAAAGTCGTTTCCCGGAGTGAGAATGGGGCACAAGCAAGCCATGATGTC
GGCCAATCCACTCGACCATCTGAACACGAGTGATCGGCAGAGGACAGCATAACGTAACGATTGAAAGGGTCCTTCGTATCGTG
CACGGTGTGGGCGTCTACTCAACCCGACTCCGCTAAGAATATCCATGGTTCGTCCTGAGAAGTCGTGCAAGCGCCTTCAACCAC
CACCATCATCAACATGGGGATCGCTAGGCTCCTACCCATATGAAACGGGATATCAGTACCCAGGCGTGTGGATACTAGGGAATC
GGGACGGACACTACAAAAGCGCTTGAAAAGTTTCTGTTCAATGTACATGGCGGTGTCGGAGGGTAAAGGGACACAGGCTGACC
GAGAGAGGGCCCCTTCATCTGTCGACATACCTATCCCAACTACGTAAGTCATATGTCTTCGCGGATACCGAAGCAGAATATCGCT
GGTTGACCTTACTATGCTACCATTTCACCTAGCCTATGGGAGCACACAAGGGACGGCGGGGACGCCATAATCTAGAGTGTGGAT
AAAACACGGGTCCCCATGCAGGGTCGAGGTCGGTCGTTATACAACCCAAGCAGTGATTTGAAGGGTCTAAGTTTCCCTAAGCAT
CCATCCTCGTAGTCGAACTCATCAATAGCATTAGATTACGCAAAGCCATGGATCCCATCTCACTAAGAACCTGCGTTTCAATATG
AACTCACAGATGAGAGCCTCTGGGATTCCCTACAGATCGTTCGCTAGTGCCATTCACTGGTAAGTCGTGAGATAGCGTTGAAGC
CACCTAGTTCGGAGAAACATCGGAACTCAGCAGGCTCGAGTTTATGACGTGCATCAAACCCAGCCTTTACTCGATTTATGACGT
GCATCGTTGTGTAGGTGAACCTGCCGTTGCAGAAGGCTTTCGCCCCCATTAATCGAGGTAATTGTACGTGTGCTGCTTCTTGCGT
AAGGTTCCGTGGCTGAGTCCAAGGAATTACTAACGCGAGCAGCGGGGCTGCTACTTGGATCAGGACTTCTGATAGGCTGGCCA
CCAGTAGCGCAGTTGTATAAACTAAAAGCCTGCGCCAGGCCTTGCTCCATGAATTCAGCGCGACGAAGGACTTCCCTACTGTTA
TTAATTTAAGCGACGAAGGACTGTACAGATGTCTCGGAATTCGTGGGGGAGTTGGAACCGGGGTGCCCCAGTGCAACTTCTTAT
GGTGGCACAACACCAAGAAAAGAAGACCCGCTGAACTGAAAATTAATGTCACTCGG

Organism	Gene	Primer name	Primers (5'→3')	Size
Trueperella pyogenes	Pyolysin	Trueperella _pyogenes_ 493bp_ploNF	AACGGCCTTCTCGACGGTTG	493 bp
		Trueperella _pyogenes _493bp_ploNR	TAGCTCGGGTCTTGTTCAGG	
		RC	CCTGAACAAGACCCGAGCTA	
	Tpyocpn60	Cpn60-f3	CGTTGAGGAGTCCAACAC	280 bp
		Cpn60-b3		
		RC	GTCAACAAGATCCGTGGC	
Chlamydia abortus	Outer_membrane_protein	ch1-omp2	ATGTCCAAACTCATCAGACGAG	587 bp
		ch2-omp2	CCTTCTTTAAGAGGTTTTACCCA	
		RC	TGGGTAAAACCTCTTAAAGAAGG	

>Bovreproseq_synthpos_True_pyo_plo_cpn60_Chlamydia_omp2
TTGTCCCGACTCCCCTTCCAACGGCCTTCTCGACGGTTGGCACTCGATGAATGCCGGAGACGCCGGCCGCGTACGTAATATACC
GGCTCAGAGAACCGTAATTGTCAGGAGCGAGGGTCGGCACAACACTATGTGAACGCGGCCGATTTATCAGTGGTGTCCTCTTT
GACACGCCAGAACACATCGTCATTTTGAGTCATACCATCTTTGTCACTTTCGCAGACGAATTGCCATTATCTTCCAGTAATGAAAG
CATTACGTATGACCATCATAGTTACCTAGGGCGCCCATGAATGAAGTGCACGCTAAGTTGTCACTGGCGGAGGGGCTGCACACT
ATCATTCACCGTAGACGGACGTCGAAAATTCTCTGGTGATGTCGTGTATGCACTACAACAAATGGTGTCCTACTACATGTGATCT
TTGCCCGTTTGATGACACCTCAGAGGGTCTCGGCTTAGGCTCCTGCCAGTGTCAATCGTCATAGGCGCCTGAACAAGACCCGAG
CTACCAGGCTCGAGAGTCTAAGACGTTGAGGAGTCCAACACTTCTGTTCCGGAGACGCTGACAGAATCCTCGGTGGGTTCGCCT
GCTGCCGAAAGAATATTATTCGCTGATGCAGATCGCACGCCAGGGCATTACCGACAGGGAAGTAAGACACTCGATCGCTGTAG
GTCCCATACGAGTATAAAACTCTCGACGCTTACGCTAGGTTTAGACCGAGCGGTCAAGTGAAGCTTCATGAAGGTGGATCCGAT
CTCTATTGCGATGCCAATTTGCAGTAACCGCTAGGTCAACAAGATCCGTGGCTGCCTGCTAGGAATTCTAGGGATGTCCAAACTC
ATCAGACGAGTCTTGGACAACTTCGGGGGTGCCTCTGTTTCAAGGAATTAGTAGTATCTGAGGAAATAGGATATTGCGCCTCCA
TTGTAAGCATGGTCGGGGCCCACCTCGGCCTGAAAGCCCCCCAATCAACGGCCAAGTACGGACGCAGCCCACTGTGGACCCGT
GAGTGAACTCATCAACAGAAAGCCTGATCAATACACACGAAAACAAAGCCACGAAGTCTATTCGATGGCCAAAATCTGAGTGAC
TATTAGTCTTGGATCGGAGCCAGTAACTTGCCAGTGTGTTGACTCGCACTTACGGTGGTACATGCTTCCCGGCAGAAAGGCTCT
CCGCGACGTTTTTGGAACAGCTCATTGCCCTAAAATGCAACCCACCTTGTAGCTAAGTACCCAAACGACTGCGAATAACTCTCGA
CTATCTATCAGGGAGATGCCCGCAATGTTATACAGTTTGCGCAAGCGCTTACAAGCCCTTAATAGTGGAGGGTGAGAGGTTCCC
AGAAACTCGGAAATCCGCCTATGTGTTGGGATCGGAGACCCATCAGACTGGGTAAAACCTCTTAAAGAAGGCGATTTCGTCGCC
CGAGTCACTACT

Organism	Gene	Primer name	Primers (5'→3')	Size
Campylobacter jejuni	Cj0414	cj0414C1F	CAAATAAAGTTAGAGGTAGAATGT	160 bp
		cj0414-R	CCATAAGCACTAGCTAGCTGAT	
		RC	ATCAGCTAGCTAGTGCTTATGG	
	hipO	HipO-F	GCTATAACTATCCGAAGAAGCCATCA	350 bp
		hipOR	GACTTCGTGCAGATATGGATGCTT	
		RC	AAGCATCCATATCTGCACGAAGTC	

>Bovreproseq_synthpos_Cjejuni_new
AATTCTATCGAATATTGACTCCACAAATAAAGTTAGAGGTAGAATGTGTCATGTACGCGAATTCTCTGGCTCAGCTTGTGTGAAT
TCGATATCCTAGGGAAAAGAGGCTCCGGCATGGGGCGGGAGCACGCCTTCAAGCCGGGACTGCTTATGGAATGGGAATCAGC
TAGCTAGTGCTTATGGGTAGAGCACGCATTACCAGAGTGCAGATTCATGTAGCTATAACTATCCGAAGAAGCCATCACGCCAGC
AATGAGCAGCCTCCGTATCCTCACACATTACTTAACAGTACTTTCAGAACCGCATTAGGTAAGGGGGTGGGCAAGTACCATGAC
CGACCAGAAGGGCACATGGTTACGAGTCCACCTACACAGGAGAGCTGGACGGAACTAAGGTTTTCACATAAGAATCGGGAGCA
AATAATCACTGTCTGTAACACAATCATCTGATCCGAATGTATTTGGAACTTATTGAGCATAAGTGCGCCGACAGATAAGCATTTC
GTGCCCTCCGTTTAATACGAGCGGCTTCTCCTAAAAAGCATCCATATCTGCACGAAGTCTATTACGACCGACAGACCTCTCTGCC
ACGGTCTCTGCCACGGTCTCGC

Organism	Gene	Primer name	Primers (5'→3')	Size
Trueperella pyogenes	superoxide dismutase	sodA F	CGAGCTCGCCGACGCTATTGCT	160 bp
		sodA R	GAGCATGAGAATCGGGTAAGTGCCA	
		RC	TGGCACTTACCCGATTCTCATGCTC	

>Bovreproseq_synthpos_Trueperella_pyo_sodA_new
CAGCTAGTTCTTACAAGACCCTTCGAGCTCGCCGACGCTATTGCTGGTTCTCCGCGACTGCGTTTGCTCGTCACCAACATACAGG
CTACCAACCTCCCGAAAAAGCTGCACTTGCCACAGTAGCTTTAGAGTACCGCTATTTTACGCGAACTAGTCATACCGTGACTCCT
AAAGTCCAGATTGGACACAGAATGCAGTGGCACTTACCCGATTCTCATGCTCAATGCAGGTCGTCCCGCCGACATGCTACTGC

Organism	Gene	Primer name	Primers (5'→3')	Size
Mycoplasma bovis	PPSM5-1	PpSM5-1	CCAGCTCACCCTTATACATGAGCGC	442 bp
		PpSM5-2	TGACTCACCATTTAGACCGACTATTTCAC	
		RC	GTGAAATAGTCGGTCTAAATGGTGAGTCA	
	uvRC	uvrC_MB-F3	CCTGTCGGAGTTGCAATTGTT	368 bp
		uvrC_MB-B3	CGGTCAACTTCAACTTGAATTTG	
		RC	CAAATTCAAGTTGAAGTTGACCG	

>Bovreproseq_synthpos_Mbovis_new
CCGACAGACCTCTCTGCCACGGCCAGCTCACCCTTATACATGAGCGCTCTCGCGCATTACGATACCCACTGTTAACTCGAAAGCA
CCGTCATCAGGCCTGATGTGAGAGATGGGAGTTCTGCATTAGTTTGTATTCACCTAACCCTGGGCTGGCTTTGCATAGCACTCTC
CTGCGCCGGCGACCAGATCCTGGTGATTTTTGACCCGCATACATATCGATACAAGTCCTACAGTTTGTTAGGGTGGCACATCGG
ACCTGCTACTGGGAGCCCCATGTATGATAGGGAATAGTAGAAGTCGGGATACTACCGGTGGGTGTTCGATGGCGAGTAGGCG
ACTCGGTAAACGTCTTGACGATCCCTTCTTATTTGTGCAATGGAAGGATTCCCTTTGCATTAACCTTCTTATTCGTCCAGCGAGAG
CACGTCGCTCCACGTGAAATAGTCGGTCTAAATGGTGAGTCAAGATCGGGCGAGGTTGTTTGACTCTAAAAGTATCAGTTCTAC
CCACTATACCTGTCGGAGTTGCAATTGTTGGATAATAAGGCGTTGAAGAGACTTAAAGCAGGATTTCTGGTTGTCTCACTTTCCA
CCCTCCTGCCGGCCGTCGTCTCTCGAGTTTTTGTTTAGGGAGCTGCCCTGTACGAAGTAAACACCGTGCCCATGTTCACTTGGAG
GCCCACGACACTAGCGTCAACGTATCCCTCTGGTTGGACTTTGTGAGCAGGGTGGTTGTACGTGCGCGAGTTGAGTTCTCAATG

TACCTGTGACAGTGGCCCCTCTGGCCAGCTTGCATTGCGGAAGTGCAGAGCGGAACGGAGCAAGTAAATGTCAAGTGCACCGA
CCTTATGTAAGTGAGTCAAATTCAAGTTGAAGTTGACCGGCGATGGTATTGACCTTGTAATGTATCACA
