# Supplementary material for: Targeted syndromic next-generation sequencing panel for simultaneous detection of pathogens associated with bovine reproductive failure
Source: J Clin Microbiol. 2024 Dec 10;63(1):e01433-24. doi: 10.1128/jcm.01433-24 (PMC11784112; doi:10.1128/jcm.01433-24)
Supplement: Figure S6 — Comparisons of sequencing depths and read counts. [file jcm.01433-24-s0002.pdf]

# Supplemental File S6: Figures

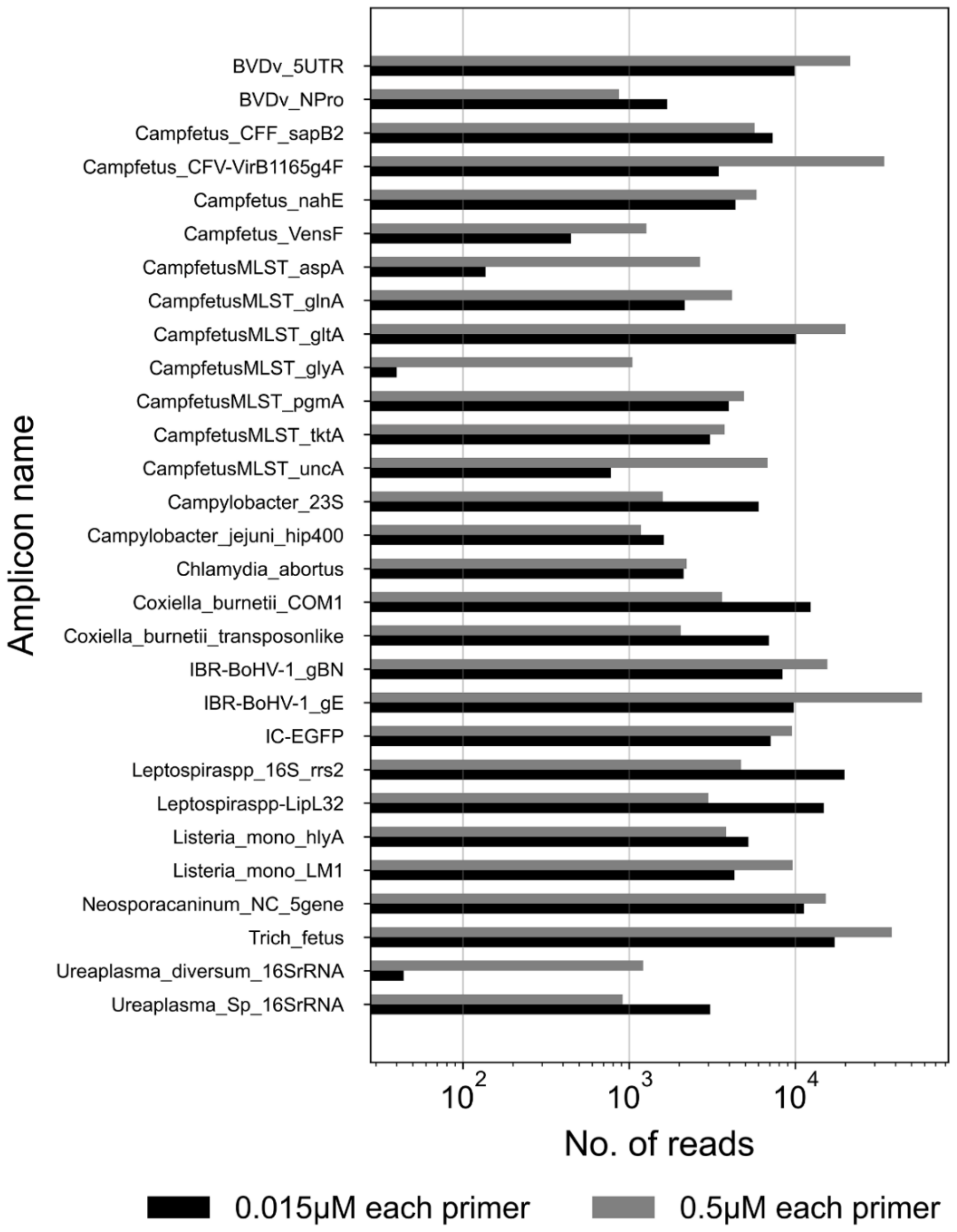


*Figure S6-1: Comparison of sequencing depth for amplicons produced with two different primer concentrations (0.015 μM and 0.5 μM) using NEB Q5 High-Fidelity 2x Master Mix and an annealing temperature of 57°C. The higher primer concentration of 0.5 μM consistently yielded greater read depths across most amplicons compared to the lower concentration of 0.015 μM. This experiment was performed without the reverse-transcription step and includes results only from synthetic controls for 12 pathogens (29 synthetic targets).*

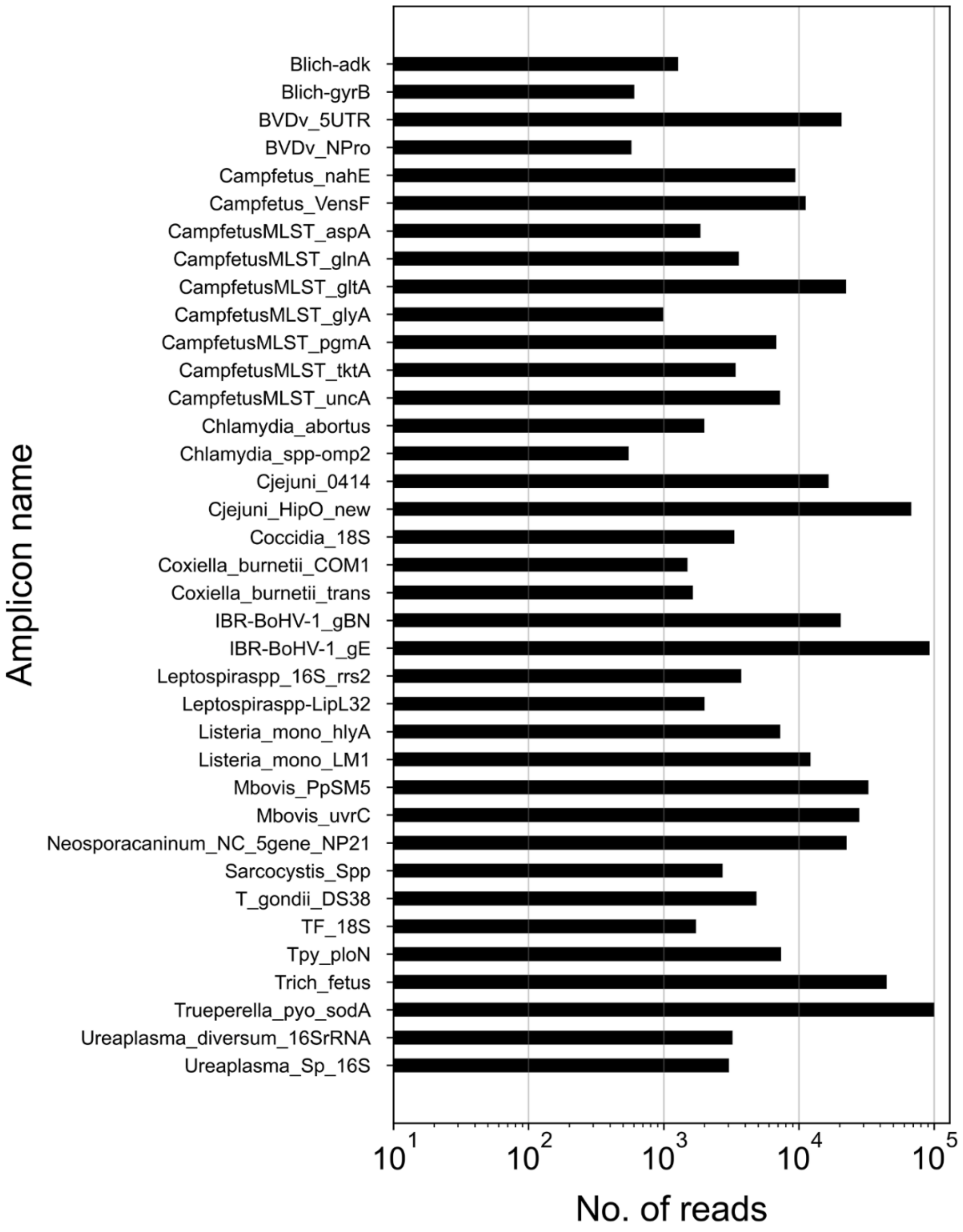


*Figure S6-2: Comparison of read counts for each synthetic control from a single PCR reaction (including reverse transcription step), where the final version of the pooled control was used as a template. All 37 synthetic targets were detected from a single PCR reaction.*
